# Phylogeography of a widespread species: pre-glacial vicariance, refugia, occasional blocking straits and long-distance migrations

**DOI:** 10.1093/aobpla/plw003

**Published:** 2016-01-14

**Authors:** Xabier Santiso, Lúa Lopez, Rubén Retuerto, Rodolfo Barreiro

**Affiliations:** 1 Área de Ecoloxía, Facultade de Bioloxía, Campus Vida, Universidade de Santiago de Compostela, 15782 Santiago de Compostela, Spain; 2 Área de Ecología, Facultad de Ciencias, Campus A Zapateira, Universidad de A Coruña, 15071 A Coruña, Spain

**Keywords:** *Arbutus unedo*, clades, cpDNA, Lusitanian, Mediterranean, refugia, strait, vicariance

## Abstract

Phylogeographic studies give us the opportunity to reconstruct the historical migrations of species and link them with climatic and geographic variation. They are, therefore, a key tool to understanding the relationships among biology, geology and history. One of the most interesting biogeographical areas of the world is the Mediterranean region. However, in this area, the description of concordant phylogeographic patterns is quite scarce, which limits the understanding of evolutionary patterns related to climate. Species with one-dimensional distribution ranges, such as the strawberry tree (*Arbutus unedo*), are particularly useful to unravel these patterns. Here, we describe its phylogeographic structure and check for concordance with patterns seen in other Mediterranean plants: longitudinal/latitudinal clines of diversity, evidence for glacial refugia and the role of sea straits in dispersal. We also identify the most likely source for the disjunct Irish population. With this aim, we sequenced four chloroplast non-coding fragments of *A. unedo* from 23 populations covering its whole distribution. We determined the genetic diversity, population structure, haplotype genealogy and time to the most recent common ancestor. The genealogy revealed two clades that separated during the last 700 ky but before the last glacial maximum. One clade occupies Atlantic Iberia and North Africa, while the other occurs in the Western Mediterranean. The Eastern Mediterranean is inhabited by newer haplotypes derived from both clades, while the Irish population is closely related to Iberian demes. The straits of Sicily and Gibraltar partially restricted the gene flow. We concluded that a vicariance event during the Late Quaternary in the western end of the species' range followed by eastward migration seems a likely explanation for the observed phylogeographic pattern. The role of straits indicates an occasional communication between Europe and North Africa, suggesting that the latter was a novel refugia. The East–West genetic split in Iberia is consistent with the refugia-within-refugia model. Finally, the strawberry tree possibly reached Ireland from Iberia instead of throughout the maritime fringe of France as previously thought.

## Abstract (precis)

The strawberry tree diverged into two groups during the Quaternary, but before the LGM, surviving in refugia located in the western end of the Mediterranean region and with the Eastern Mediterranean being colonized more recently. This migration was possible because Europe and North Africa were occasionally connected through the straits of Gibraltar and Sicily. Likewise, our evidence supports arrival in Ireland from northern Iberia in post-glacial times. Altogether, our results reveal the considerable ability of the strawberry tree for dispersal, allowing it to migrate over thousands of kilometres and cross stretches of sea, which may be crucial for its future survival.

## Introduction

Phylogeography uses molecular information to infer the historical forces that have shaped the genealogical architecture of populations and closely related species (Avise 2009). The typical way to do so is by reference to the framework of genealogical concordance (Avise and Ball 1990). In particular, multispecies concordance has stimulated a number of comparative phylogeographic surveys for faunas and floras with mixed success (Avise 2004). One region where the description of multispecies concordant patterns is particularly difficult is the Mediterranean basin, one of the earth's 25 biodiversity hotspots, with 4.3 % of the world's 300 000 plant species (Myers *et al*. 2000). The basin serves as a contact zone for three continents separated by a tortuous sea, resulting in a very heterogeneous region in terms of climate and geography (Quézel and Médail 2003), which is mirrored by an intricate phylogeography. Thus, a recent review of the phylogeographic studies conducted in the region concluded that, compared with the Alps or North America, the Mediterranean lacks largely common patterns across plant species (Nieto Feliner 2014). Moreover, the phylogeographic complexity of the region might also be a consequence of a blurring of genetic footprints over time. Thus, the less drastic effects of the Pleistocene glaciations in the Mediterranean area are thought to have facilitated an accumulation of species responses to the successive palaeoenvironmental changes (Migliore *et al*. 2012).

Despite the above, commonalities still exist, even though they frequently show some inconsistencies. Similarly to other areas of the world, there is a south–north decrease in genetic diversity (Hewitt 2000). This latitudinal cline must have been caused by a leading-edge expansion from southern refugia, and many studies have corroborated the role of the three large Mediterranean peninsulas (Iberia, Italy and Balkans) as refugia for the survival of species and engines of speciation (Hewitt 2000, 2011). Moreover, it is now widely recognized that the ‘refugia-within-refugia’ model (Gómez and Lunt 2007) explains the phylogeographic breaks identified within these peninsulas, particularly in Iberia (López de Heredia *et al*. 2007; Rodríguez-Sánchez *et al*. 2010). Nonetheless, it is growingly acknowledged that refugia were not confined to major peninsulas and many of them have been identified in areas that were previously attributed a lesser role (large islands, North Africa, Turkey and Catalonia-Provence). Again, this complex arrangement of refugia possibly evidences the cumulative effects of historical and environmental factors that occurred since the Tertiary (Médail and Diadema 2009).

Another interesting pattern is the finding that genetic diversity increases from west to east along the Mediterranean in many species (Conord *et al*. 2012). Several Mediterranean trees even show a clear division between the eastern and the western ends of the Mediterranean, with eastern lineages commonly pre-dating western ones (Lumaret *et al*. 2002; Rodríguez-Sánchez *et al*. 2010). These longitudinal patterns have been attributed to relatively recent processes such as stronger demographic impacts under local last glacial maximum (LGM) climate in west populations and an east–west recolonization during the Holocene (Conord *et al*. 2012). Alternatively, the east–west vicariance has also been interpreted as a genetic footprint of much older, pre-Quaternary range dynamics (Petit *et al*. 2005; Médail and Diadema 2009; Rodríguez-Sánchez *et al*. 2009, 2010). Finally, the effectiveness of the sea straits (like Gibraltar or Sicily) as phylogeographic barriers has varied over time given the eustatic sea-level shifts during the Pleistocene (Hewitt 2000) and even the same strait (e.g. Gibraltar) served as barrier or not depending on the species (Fernández-Mazuecos and Vargas 2010; Hewitt 2011).

**Figure 1.**
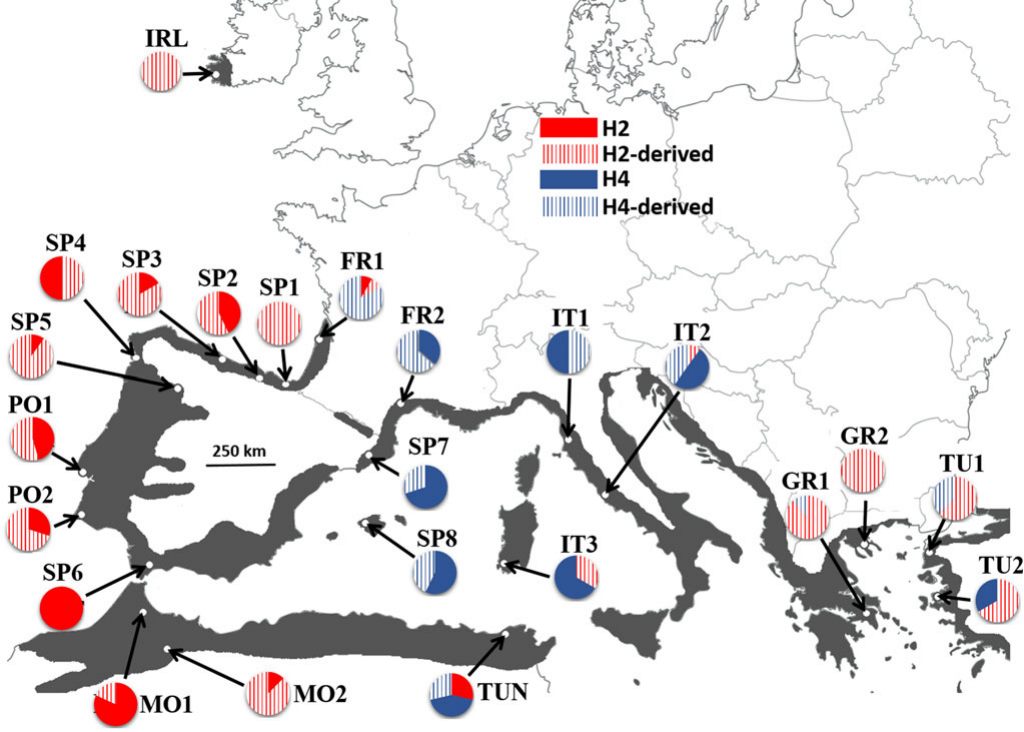
Population frequencies of the cpDNA lineages across the range (greyed) of *A. unedo*.

Given its phylogeographic complexity, the study of simplified systems such as organisms with one-dimensional distribution range may help in the search for congruence in the Mediterranean basin (Clausing *et al*. 2000; Nieto Feliner 2014). This is the case of *Arbutus unedo* (strawberry tree), a small tree with a neat circum-Mediterranean distribution (Sealy 1949; Torres *et al*. 2002) that occupies a narrow coastal fringe from Tunisia to Morocco and from Spain to Turkey (Fig. 1). From a historical perspective, Axelrod (1975) cited the genus *Arbutus* as a component of the Madrean–Tethyan flora that existed between western North America and Eurasia until the end of the Oligocene (25 Mya). The divergence between Mediterranean and North American *Arbutus* was estimated in at least 21.2 Mya (Hileman *et al*. 2001). The ancestral *A. unedo* remained in the area currently occupied by Hungary–Poland–Bulgaria until the Messinian (Palamarev 1989), *A. andrachne* being the closest relative. Interestingly, the strawberry tree also lives outside the Mediterranean bioclimatic region along the Atlantic coasts of Morocco, Iberian Peninsula, France and Southwest Ireland. In the Atlantic, the strawberry tree shows the discontinuous distribution typically found in members of the ‘Lusitanian flora’, a group of species that have puzzled biogeographers because they occur in Ireland and northern Iberia while they are largely absent from intervening countries (Sealy 1949; Beatty and Provan 2013). Early interpretations viewed Lusitanian species as relicts of the Tertiary flora that survived through the glacial period (Sealy 1949; Webb 1983), but a post-glacial colonization of Ireland from southern refugia is currently considered a more likely alternative. Accordingly, Iberia has been described as the likely origin for Irish oaks (Kelleher *et al*. 2004; Lowe *et al*. 2005), whereas the precise location of the putative southern refugia remains elusive for other species (Beatty and Provan 2013, 2014). Some Lusitanian species may even have recolonized Ireland with human help rather than naturally (Foss *et al*. 1987; Smith and Waldren 2010). In the particular case of the strawberry tree, its presence in Ireland since at least 4000 years ago has been confirmed by both pollen (Mitchell 1993) and wood remains (Van Rijn 2004). Accordingly, it might have reached Ireland in the mid-Holocene thermal optimum, 9000–6000 years ago (Davis *et al*. 2003; Peyron *et al*. 2012).

In this study, we sequenced four chloroplastidial non-coding fragments to address a number of specific issues: (i) to estimate the time to the most recent common ancestor (TMRCA) of *A. unedo* and to infer possible colonization routes along the Mediterranean and the Atlantic façade, (ii) to test whether some patterns detected in other Mediterranean plants also apply to the strawberry tree (longitudinal/latitudinal clines of diversity, placement of glacial refugia and role of the straits as barriers to dispersal) and (iii) to identify the likely source for the disjunct Irish population. To our knowledge, this is the first range-wide study of the phylogeographic structure of *A. unedo*.

## Methods

### The species

*Arbutus unedo* is a small–medium tree from the family Ericaceae, subfamily Arbutoideae. Genus *Arbutus* includes several members in USA and four in Europe: *A. andrachne*, *A. pavarii*, *A. canariensis* and *A. unedo* (Torres *et al*. 2002). Flowering spans from September to December and fecundation is entomophilus (Mitchell 1993). Fruits take 12 months to ripen and seeds are dispersed mainly by birds (Sealy 1949). Regarding soil conditions, the strawberry tree prefers siliceous or decarbonated substrata, although it also occurs on formations of carbonated substrata, on heavy clay soils, sandstone and limestone, with pH ranging from 4 to 7 (Sealy 1949; Torres *et al*. 2002). The distribution of the species is limited by a mean January temperature of 40 °F (4.4 °C) (Sealy 1949).

### Sample collection and DNA extraction

The sampling plan covered the whole range of *A. unedo* and included leaf samples from 23 wild populations in nine countries (Table 1). Additionally, we collected samples from two individuals of *A. andrachne*, the closest relative of *A. unedo*, from Çanakkale (Turkey). At each population, leaves were collected from 12 adult trees separated at least 15 m from each other to minimize the risk of sampling relatives. Leaves were wrapped in Kimtech Science wipes (Kimberly-Clark Europe Ltd, UK) and stored *in silica* gel until DNA extraction. DNA was extracted with the Realpure Genomic DNA extraction kit (REAL) according to manufacturer’s instructions except that we extended the incubation times for cell lysis (2 h at 55 °C) and protein precipitation (20 min at −20 °C). Extractions were conducted in batches of 23 samples plus a negative control; each batch combined individuals from at least five populations. The quality of extracted DNA and negative controls was checked on 1.5 % agarose gels.

**Table 1.** *Arbutus unedo* sampling localities, UTM coordinates, sample size (*n*), number of segregating sites (*S*), number of total (Ht) and private (Hp) haplotypes, haplotype (*h*_d_) and nucleotide (*π* × 100) diversities (with SD) obtained based on the analysis of sequences with non-coding cpDNA fragments. Bold values indicate co-occurrence of haplotypes separated one large indel (36 or 37 bp), respectively.

Population	ID	Position	*n*	*S*	Ht	Hp	*H*_d_	*π* × 10^2^
Killarney (Ireland)	IRL	29U 465211 5763297	12	1	2	0	0.530 (0.077)	0.022 (0.02)
Bordeaux (France)	FR1	30T 643276 4939413	12	6	4	1	0.455 (0.170)	**0.631 (0.34)**
Montpellier (France)	FR2	31T 531757 4829416	11	3	4	1	0.764 (0.083)	0.047 (0.04)
Botaleku (Spain)	SP1	30T 573213 4792097	6	2	3	0	0.733 (0.155)	**0.812 (0.49)**
Balmaseda (Spain)	SP2	30T 485165 4778410	7	3	4	0	0.810 (0.130)	**0.457 (0.27)**
La Franca (Spain)	SP3	30T 372037 4805531	6	2	3	1	0.600 (0.215)	0.172 (0.11)
Tomiño (Spain)	SP4	29T 515032 4641671	6	2	2	0	0.600 (0.129)	0.049 (0.42)
Ponferrada (Spain)	SP5	29T 705440 4722721	10	2	3	0	0.378 (0.181)	**0.303 (0.18)**
Malaga (Spain)	SP6	30S 376766 4076446	12	0	1	0	0.000 (0.000)	0.000 (0.00)
Montseny (Spain)	SP7	31T 454080 4620578	10	3	3	0	0.511 (0.164)	0.170 (0.11)
Esporles (Spain)	SP8	31S 461243 4392754	7	3	4	1	0.714 (0.181)	**0.934 (0.54)**
Arrabida (Portugal)	PO1	29S 497741 4257559	11	3	4	2	0.709 (0.099)	**0.358 (0.20)**
Monchique (Portugal)	PO2	29S 538873 4126983	10	1	2	0	0.467 (0.132)	**0.689 (0.38)**
Tanger (Morocco)	MO1	30S 267001 3940810	11	2	2	1	0.327 (0.153)	0.134 (0.08)
Debdou (Morocco)	MO2	30S 492658 3759875	8	4	5	4	0.786 (0.151)	0.196 (0.12)
Orciatico (Italy)	IT1	32T 637192 4811775	8	2	2	0	0.571 (0.094)	**0.867 (0.49)**
Roma (Italy)	IT2	33T 279753 4619932	10	3	3	1	0.644 (0.101)	0.039 (0.03)
Cagliari (Italy)	IT3	32S 491121 4333556	9	6	3	0	0.556 (0.165)	**0.850 (0.47)**
Kroumerie (Tunisia)	TUN	32S 470929 4061547	7	6	4	0	0.810 (0.130)	**0.816 (0.47)**
Atenas (Greece)	GR1	34S 746586 4206313	9	5	3	0	0.639 (0.126)	**0.387 (0.22)**
Sithonia (Greece)	GR2	34T 739962 4452294	8	3	3	1	0.464 (0.200)	0.031 (0.03)
Cannakal (Turkey)	TU1	35T 459279 4441142	8	5	3	1	0.750 (0.097)	**0.901 (0.51)**
Izmir (Turkey)	TU2	35S 458841 4254352	9	6	3	0	0.639 (0.126)	**0.850 (0.47)**
Total			207	16	28	14	0.901 (0.01)	1.238 (0.60)

### Sequencing

After reviewing previous phylogeographic studies, initial trials were conducted with five non-coding cpDNA regions commonly used with trees and, specifically, in studies of closely related species: trnS(GCU)-trnG(UUC), trnH(GUG)-psbA, trnL(UAG)-rpl32, trnT(UGU)-trnL(UAA) and Intrón L (Taberlet *et al*. 1991; Shaw *et al*. 2005, 2007). These initial trials revealed a widespread occurrence of illegible sequences due to long poly-A fragments in non-coding regions (polymerase slippage). Sequencing problems were solved and clear sequences were obtained for four out of the five non-coding regions, all except trnS(GCU)-trnG(UUC), by using a high-fidelity polymerase (Phusion High-Fidelity ADN Polymerase, Thermo Scientific) (Fazekas *et al*. 2010). Polymerase chain reactions (PCRs) were performed using 10 µL of 2× Phusion Master Mix (with 1 U of Phusion High-Fidelity ADN Polymerase), 0.5 µmol L^−1^ of each primer and 1 µL of DNA (diluted 1 : 10). Amplification conditions were 30 s at 98 °C for DNA denaturation; 30 cycles of 10 s at 98 °C, 20 s at the specific annealing temperature of each primer and 30 s at 72 °C; and a final extension of 7 min at 72 °C. Annealing temperatures were 60.8 °C (trnT-trnL), 59.7 °C (trnL-rpl32), 61.8 °C (Intron L) and 66.8 °C (trnH-psbA). Before sequencing, PCR products were checked on 1.5 % agarose gels and purified with 1 µL of Exonuclease I (20 U µL^−1^) and 2 µL of FastAP (10 U µL^−1^) (Fermentas, Waltham, MA, USA). Purified PCR products were sequenced under BigDye Terminator cycling conditions on an Automatic Sequencer 3730XL (Applied Biosystems, USA). The program Geneious R6 v.6.1.4 was used to check the quality of the chromatograms and to perform alignments. Singleton polymorphisms and unique haplotypes (i.e. detected in one single individual) were corroborated with reverse sequencing reactions to discard sequencing artefacts. As the non-recombinant nature of cpDNA makes it equivalent to a single-locus marker, sequences from the four fragments were combined into a single haplotype for every individual. These sequences can be obtained from GenBank using the accession numbers: BankIt 1868954: KU194976–KU195222 (Intron L fragment); BankIt 1874447: KU205359–KU205584 (trnT-tnrL fragment); BankIt 1874452: KU205585–KU205820 (trnL-rpl32 fragment); BankIt 1874463: KU205821–KU206025 (trnH-psbA fragment). It is noteworthy that the identification of each population is represented by the last two letters and two numbers of the sequence definition name. **Supporting Information—Table S1** shows the equivalence between the population codes used in this article and the population codes that identify the populations in GenBank. Still, the identity of each fragment was retained when we calculated the TMRCA to allow for differences in the mutation rate of each region.

### Data analysis

Haplotypes were identified with DnaSP v.5 (Librado and Rozas 2009), which also provided estimates for haplotype diversity (Hd) (Nei 1987). Genetic diversity was evaluated as nucleotide diversity (*π*) using Arlequin 3.5 (Excoffier and Lischer 2010). Arlequin was also used to carry out an analysis of molecular variance (AMOVA) to assess population structure and to calculate the Fu's *F*_S_ test to detect any evidence of demographic expansion (Fu 1997). Population structure was further investigated by defining clusters of genetically similar populations with a spatial analysis of molecular variance (SAMOVA) (Dupanloup *et al*. 2002). Spatial analysis of molecular variance group populations into a user-defined number of groups (*K*) using a simulated annealing procedure to maximize the variance (*F*_CT_) among those groups. The analysis was performed in SAMOVA 2.0 (Dupanloup *et al*. 2002) running 10 000 iterations from 100 initial conditions, testing *K* = 2–9 with and without constraint for the geographic composition of the groups. The final number of groups was chosen for which *F*_CT_ began to plateau. In addition, Permut 1.0 was used to estimate population differentiation by calculating *G*_ST_ and *N*_ST_ which are two measures of populational differentiation, *G*_ST_ use only of the allelic frequencies and *N*_ST_ also take into account the similarities between the haplotypes. The analysis was performed under the assumption that a significantly higher *N*_ST_ value suggests the existence of phylogeographic structure (Pons and Petit 1996).

The genealogy of the haplotypes was inferred with the median-joining network algorithm and maximum parsimony calculation implemented in Network 4.6 (Bandelt *et al*. 1999; Polzin and Daneshmand 2003). We ran the program with *ε* = 0 and mutations weighted following recommended guidelines: 10 as a default value, 5 for very common mutations (where the various alternative stages occurred in similar proportions) and 20 for indels. To improve the interpretation of the resulting tree, we removed the resulting loops, but always without modifying the interpretation. Complementarily, we estimated the genealogy using TCS 1.21 (Clement *et al*. 2000), setting the connection limit at 95 % and considering the gaps (missing value) as fifth state.

Estimates of the TMRCA were conducted with the 207 *A. unedo* sequences in BEAST 2.1 (Bouckaert *et al*. 2014) by combining two searches with 50 million Markov chain Monte Carlo (MCMC) each and a sample frequency of 1000. The first 5 million generations (10 %) were discarded as a burn-in. We used jModelTest 2.1 (Darriba *et al*. 2012) to determine with Akaike and the Bayesian information criteria the simplest model of sequence evolution that best fitted our data. The four cpDNA fragments were tested both separately and concatenated into a single sequence. As F81 and HKY were consistently identified as best models, we used the model HKY available in BEAST. A Coalescent Constant Population was employed as tree prior and we implemented the strict molecular clock recommended for intra-species analysis (no expected rate variation among branches). Given the wide variation of substitution rates seen among flowering plants (Smith and Donoghue 2008; Huang *et al*. 2012), and considering that the mutation rate in the literature for the cpDNA fragments used in our study was 1.0^−9^ (Huang *et al*. 2012), we intentionally specified a broad prior using an uniform distribution with bounds 1.0^−10^ and 1.0^−8^ (substitutions site^−1^ year^−1^), which allowed BEAST to obtain the precise mutation rate. The four cpDNA fragments were designed as unlinked partitions to allow for variable mutation rates. Chain convergence was assessed with Tracer 1.6 (available in the BEAST2 package at http://beast.bio.ed.ac.uk/) and by checking the effective sampling size (ESS) values. Trees were summarized in a maximum clade credibility tree obtained in TreeAnotator 2.1 and visualized in FigTree 1.4.1 (both also available in the BEAST2 package).

The calculation of the TMRCAs for the groups later detected was based on independent running of BEAST, by combining two searches with 10 million MCMC, using only the haplotypes assigned to each group.

### Climatic reconstruction

The climatic reconstruction observed in Fig. 3 took as reference a couple of maps created thanks to the ‘stage 3 project’ (Van Andel 2002). The first was the map ‘tmin_21k’, which represents the seasonal minimum air temperature 21 000 years ago. The second was the map ‘tmin_mod’, which describes the current seasonal minimum air temperature. Both maps are available at ftp://ftp.essc.psu.edu/pub/emsei/pollard/Stage3/PLOT/.

## Results

### Genetic diversity and population structure

We produced sequences for 207 individuals of *A. unedo* (Table 1) plus the two individuals of *A. andrachne*. The four non-coding fragments revealed polymorphisms along the range of *A. unedo* and were retained for phylogenetic analyses. Once concatenated, and depending on the occurrence of indels, the four fragments resulted in sequences 2401–2449 bp long, equivalent to 1.6 % of the chloroplast genome of *A. unedo* (Martínez-Alberola *et al*. 2013). The data set of *A. unedo* contained 16 polymorphic sites that included 7 point mutations and 9 indels. Three indels were 1 bp long, two indels were 9 bp long and the remaining four indels were 10, 11, 35 and 36 bp long, respectively.

A total of 28 haplotypes were found **[see** **Supporting Information—Fig. S1****]**, and half of them (14) were private (Hp); interestingly, five Hp were detected in North Africa (Morocco). The two individuals of *A. andrachne* produced a single haplotype that differed by 16 new point mutations from the consensus sequence of *A. unedo*. Haplotype diversity in *A. unedo* was 0.901 (±0.01) (standard error), while nucleotide diversity (*π*) (×10^2^) was 1.238 (±0.60). Nonetheless, genetic diversity was unevenly partitioned among populations as Hd and *π* estimates for the 23 populations ranged 0.000–0.810 and 0.000–0.934, respectively (Table 1). The highest *π* values were consistently recorded in populations that contained a mixture of haplotypes separated by long indels (35 or 36 bp).

Total gene diversity (ht) was 0.919 (±0.02), while the average within-population gene diversity (hs) was 0.585 (±0.04). We also calculated the diversity at a regional scale by partitioning the populations into three groups suggested by the phylogeographic analyses (see below): Eastern Mediterranean (GR1, GR2, TU1 and TU2), Western Mediterranean (FR1, FR2, SP7, SP8, IT1-IT3 and TUN) and Atlantic coast (IRL, SP1-SP6, PO1, PO2, MO1 and MO2). Diversity at regional scale increased from the Eastern (Ht = 7, Hd = 0.693 ± 0.076, Π × 10^2^ = 0.58 ± 0.30) to the Western Mediterranean (Ht = 15, Hd = 0.762 ± 0.044, Π × 10^2^ = 0.63 ± 0.32), and to the Atlantic coast (Ht = 13, Hd = 0.787 ± 0.029, Π × 10^2^ = 0.75 ± 0.38).

The AMOVA revealed that 38.8 % of the genetic variation was due to differences between populations, and the resulting *F*_ST_ value was highly significant (*P* < 0.001). *N*_ST_ (0.645 ± 0.067) was significantly higher than *G*_ST_ (0.363 ± 0.040) (*P* < 0.001) indicating the existence of a strong phylogeographic structure (Pons and Petit 1996). Furthermore, SAMOVA showed that *F*_CT_ began to plateau at 0.697 for three groups of populations regardless of the alternative used (with or without constraint for the geographic composition of the groups) (Table 2). One group was geographically homogeneous and included the populations sampled in the Eastern Mediterranean plus Bordeaux (FR1) in the Atlantic France. Another group was split into two sets of non-adjacent populations because populations from Atlantic Iberia–North Africa grouped with populations from Western Mediterranean. Finally, a third, smaller group clustered the Irish population with two locations in Atlantic Iberia. Less than 8 % of the variation was among populations within groups and nearly 23 % was within populations.

**Table 2.** Results of the SAMOVA for the number of groups (*K* = 3) for which *F*_CT_ reached a plateau. ***P < 0.001.

Source of variation	df	MS	Variance components	Percentage of variation	Fixation index
Among groups	2	1891.700	14.6553	69.73	*F*_CT_ = 0.697***
Among populations	20	373.372	1.5597	7.42	*F*_SC_ = 0.245***
Within populations	184	883.673	4.8026	22.85	*F*_ST_ = 0.771***
Group composition	1: FR1, FR2, SP7, SP8, IT1, IT2, IT3, TUN 2: SP1, SP2, SP3, SP4, SP6, PO1, MO1, MO2, GR1, GR2, TU1, TU2 3: IRL, SP5, PO2				

### Haplotype genealogy and distribution

**Figure 2.**
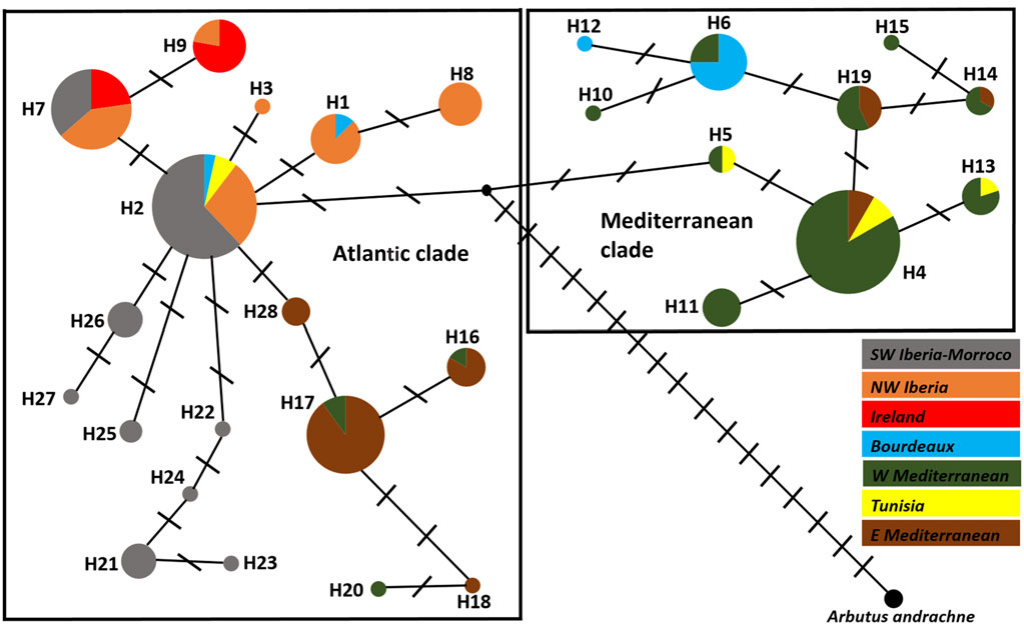
Median-joining haplotype network for 18 *A. unedo* non-coding cpDNA haplotypes with *A. andrachne* as external rooting. Each circle represents a haplotype (name outside) while colours indicate sampling regions (SW Iberia–Morocco = PO1, PO2, SP6, MO1 and MO2; NW Iberia = SP1–SP5; Ireland = IRL; E France = FR1; W Mediterranean = FR2, SP7, SP8 and IT1–IT3; Tunisia = TUN; W Mediterranean = GR1, GR2, TU1 and TU2). Circle size is proportional to haplotype frequency. Thick solid lines delineate two main clades (Atlantic and Mediterranean) separated by the inferred ancestral node (root; solid black dot). Short transversal lines are mutational steps.

Haplotypes H2 and H4 dominated the data set (37 %), whereas 12 haplotypes were very rare, as they were detected in just one (10 unique haplotypes) or two individuals (2 haplotypes) **[see** **Supporting Information—Table S1****]**. The median-joining network (Fig. 2) yielded a genealogy where many haplotypes were just one mutational step away from their closest relative. The topology of the network also revealed two clades separated from the ancestral node inferred with the help of *A. andrachne* by two mutational steps, and from each other by four mutational steps. One clade was dominated by H2 at the centre of 17 haplotypes arranged in a star-like pattern (henceforth H2-derived), while the other clade was dominated by H4, again surrounded by 9 less-common haplotypes (H4-derived).

The partition of haplotypes in two large lineages was remarkably consistent with their geographical location as well as with the clusters of populations identified by SAMOVA (Fig. 2). Accordingly, the dominant haplotypes H2 and H4 were regionally segregated and their ranges barely overlapped because H4 was detected only in the Mediterranean, while plants assigned to H2 came from sites along the Atlantic and North Africa. Kroumerie (TUN) in North Africa was the only site where H2 and H4 were found living in sympatry. Like H2, most of the H2-derived haplotypes occurred along the Atlantic from Ireland to Morocco. In fact, H2 and H2-derived sequences (henceforth the Atlantic clade) were the only haplotypes detected in populations from the Atlantic shoreline other than FR1 (see below). Interestingly, however, another subset of five closely related H2-derived sequences had a non-Atlantic provenance as they belonged to plants sampled at Sardinia (IT3), Rome (IT2) and, particularly, the Eastern Mediterranean (Greece and Turkey). In comparison, no haplotype from the Atlantic clade was ever found in the NW Mediterranean (NE Spain, W France and N Italy). The H4-derived haplotypes (Mediterranean clade) also resembled the geographical distribution seen in their ancestral haplotype H4 as they were largely confined to the Mediterranean basin. The exceptions were two H4-derived haplotypes (H6 and H12) that eventually reached a small section of the Atlantic species' range in W France (FR1), where they lived with congeners from the Atlantic phylogroup. Interestingly, the ancestral haplotypes H2 and H4 seemed largely absent from the populations sampled at the northern (Ireland) and eastern (Greece and Turkey) edges of the species' range; only H4 was detected in three individuals from Turkey (population TU2). Additionally, we analysed the trees obtained with TCS **[see** **Supporting Information—Fig. S2****]** and BEAST **[see** **Supporting Information—Fig. S3****]**. The previous description in two sublineages was corroborated and also the distribution of the main haplotypes. Even if the general patterns were concordant, some exceptions were found. TCS assigned H11 and H10 to the sublineage H2, while H20 was placed into sublineage H4 and it placed H7 and H9 as a different group, although closest to H2 than to H4 (that was checked with complementary analysis) (not shown). Likewise, BEAST analysis located H15 into sublineage H2, while H18 and H20 were placed into sublineage H4. Despite these minor modifications, the previous interpretation of the Network tree is still consistent.

### Timing of the diversification

Initial trials in BEAST were run using exponential growth as the coalescent tree prior. Regardless of the data set tested (complete data set, Atlantic populations, Mediterranean populations, individuals from the Atlantic clade or individuals from the Mediterranean clade), the posterior distribution of the growth rate [95 % highest posterior density (HPD)] always included zero indicating no support for the existence of exponential growth and establishes the plausibility of the constant population growth model, based on our data. The absence of demographic expansion was confirmed by Fu's *F*_S_ tests run for the complete data set and for each genetic clade treated separately (*P*-value always >0.65). Therefore, the final estimates of TMRCA relied on a constant growth model. However, the presence of 11 out of the 14 private haplotypes in the Atlantic clade might indicate a higher ability to diverge and spread. The latter is further supported by the higher number of haplotypes and more extensive geographical distribution of this clade. The Bayesian tree inferred by BEAST reproduced the neat division in two clades (Atlantic and Mediterranean) found in the network analysis **[see** **Supporting Information—Fig. S2****]**. The BEAST inference suggested that the split between Atlantic and Mediterranean lineages must have occurred before the LGM, around the Middle/Late Pleistocene boundary. Thus, the TMRCA for *A. unedo* was 365 ky (95 % HPD: 48–859 ky; effective sample size, ESS = 1295). The calculation of the TMRCAs for the Atlantic and the Mediterranean lineages was 258 ky (95 % HPD: 43–585 ky, ESS = 804) for the Atlantic and 194 ky (95 % HPD: 9–497 ky, ESS = 596) for the Mediterranean.

## Discussion

### East–West pattern

The most conspicuous attribute in our data set is the separation of the strawberry tree into two neat matrilineal lineages with longitude. East–west phylogeographic breaks seem common in the Mediterranean, and extreme cases include plants with disjunct populations on both ends of the Mediterranean (Kadereit and Yaprak 2008; Casimiro-Soriguer *et al*. 2010; Nieto Feliner 2014). In plants, however, most examples involve species with continuous ranges that still show a phylogeographic split between the Eastern and the Western Mediterranean (Lumaret *et al*. 2002; Hampe *et al*. 2003; Rodríguez-Sánchez *et al*. 2009; Escudero *et al*. 2010). The peculiarity in the strawberry tree is that both lineages appear to have originated in the western end of Mediterranean region: one in the Atlantic coasts of Iberia and North Africa, and the other in the Mediterranean basin itself. The presence of several genetic clades in the Western Mediterranean has been observed in other plants and was linked to range contractions during the Quaternary glaciations (Kadereit *et al*. 2005; Lumaret *et al*. 2005). In the case of *A. unedo*, we infer that the diversification in two lineages also occurred in the late Pleistocene but before the LGM, suggesting that it may have coincided with the hardest glaciations recorded in the Quaternary (Médail and Diadema 2009; Stewart *et al*. 2010). Moreover, the strawberry tree possibly survived the late Quaternary in the Western Mediterranean given the observation that the ancestral haplotypes H2 and H4 are largely restricted to this region. In this regard, several areas that had previously been attributed a lesser role are now considered to have played an important role as glacial refugia (Médail and Diadema 2009). Interestingly, the latter include several areas located in the Western Mediterranean (large Mediterranean islands, North Africa and Catalonia) that may also have played a role in the case of *A. unedo*.

**Figure 3.**
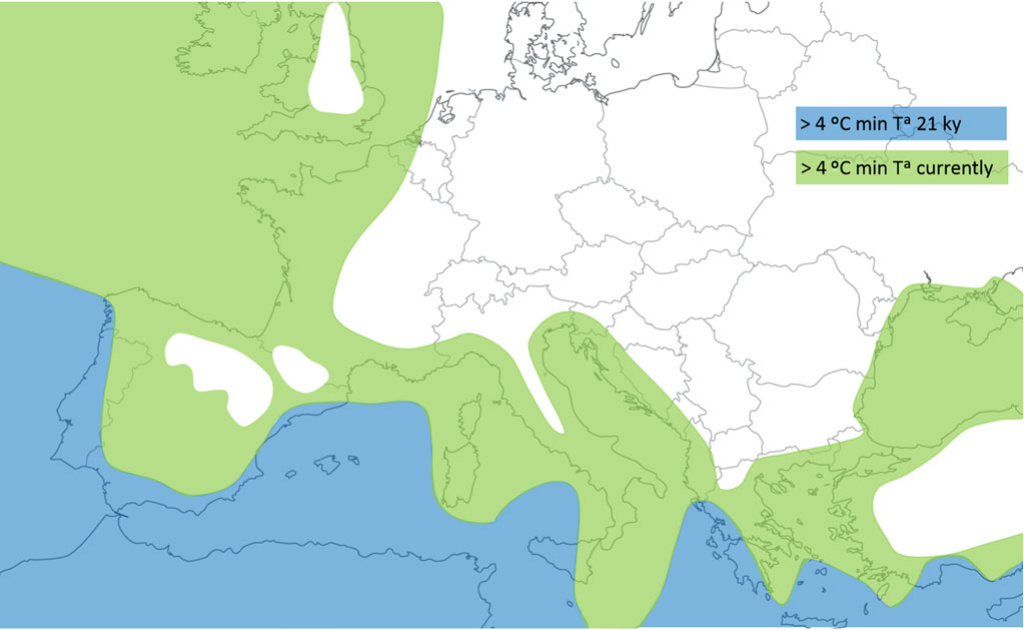
Areas where the minimum monthly temperatures suitable for *A. unedo* (>4 °C) occurred: during the LGM (21 ky, blue) and in modern times (green).

The more recent colonization of the Eastern Mediterranean by the strawberry tree contrasts with most studies of plants where eastern lineages typically are more ancient (Lumaret *et al*. 2002; Kadereit and Yaprak 2008; Escudero *et al*. 2010; Conord *et al*. 2012; Migliore *et al*. 2012). In trees, the predominance of westward migrations has been attributed to divergence that predates the Pleistocene (Lumaret *et al*. 2002, 2005; Petit *et al*. 2005). Alternatively, however, genetic and fossil evidence linked the East–West break to range fragmentations and to more severe climate in the west during the Pleistocene (Escudero *et al*. 2010; Migliore *et al*. 2012). In comparison, there are few examples of colonization in the opposite direction. Still, North African populations were likely candidates for the ancestral pool of *Europhaca* (Casimiro-Soriguer *et al*. 2010), while the submediterranean alpine *Anthyllis montana* also migrated eastward from Iberia up to the Balkans along the northern edge of the Mediterranean (Kropf *et al*. 2002). Similarly, *Pinus pinaster* (Bucci *et al*. 2007) and *Erica arborea* (Désamoré *et al*. 2011) seem to have migrated eastward along North Africa to Europe. Nonetheless, the closer example to the case of the strawberry tree is *Myrtus communis*, another circum-Mediterranean shrub with fruits dispersed by birds. Following differentiation events during the Pleistocene, some matrilineal lineages of *M. communis* spread from west to east, in particular, to the Balkan Peninsula where they met with older lineages (Migliore *et al*. 2012). In *A. unedo*, an eastward colonization would seem consistent with (i) a predominance of younger haplotypes in this area (H2-derived and H4-derived) and (ii) the likely unsuitability of the West Mediterranean during glacial maxima. Accordingly, paleoclimatic reconstructions of the LGM indicate that minimum monthly temperatures in the Aegean–Anatolia area were below the 4 °C limit required by the strawberry tree for survival (Sealy 1949) (Fig. 3).

### The role of the straits

The likely route for the movement of the Atlantic lineage towards the Eastern Mediterranean was through North Africa to mainland Italy and the Balkans by crossing the Strait of Sicily. The latter has been involved in biogeographical connections for many taxa. In trees, a crossing by matrilineal lineages from North Africa has been inferred for some species (Lumaret *et al*. 2005; Désamoré *et al*. 2011; Migliore *et al*. 2012) but not for others (Hewitt 1999; Lumaret *et al*. 2002). Nonetheless, the strait still had an impact on the migration of the Atlantic clade of *A. unedo* because only a handful of H2-derived haplotypes managed to reach the Eastern Mediterranean, contrarily to other trees where several haplotypes were common to both sides of the strait (Lumaret *et al*. 2005; Migliore *et al*. 2012). Our estimate of the TMRCA for the Atlantic clade suggests that the strawberry tree crossed the Strait of Sicily in the last 200 000 years but still before the LGM. Interestingly, the impact of the strait on the migration of the Mediterranean lineage was different because the same set of haplotypes occurs on both sides of the strait. However, the Mediterranean clade seemed unable to spread beyond Tunisia, a confinement that would be consistent with the ‘high density blocking hypothesis’ (Hewitt 1999) if North Africa was already colonized by the Atlantic lineage before the arrival of the Mediterranean clade.

Gibraltar, the other large strait in the region, also had partial success in interrupting the gene flow of *A. unedo*. Only plants with Atlantic ancestry live on both sides of the strait, and the ancestral haplotype H2 is equally common to the north and to the south of Gibraltar. However, none of the H2-derived haplotypes recorded in Iberia occurs in North Africa and the other way around. Hence, the isolation of the populations on both sides of the strait must have lasted long enough to allow the diversification of distinct sets of haplotypes on each area, such as many of the private haplotypes, supporting the role of North Africa as a refugium for *A. unedo*. Interestingly, Rodríguez-Sánchez *et al*. (2008) noted that colonization ability, rather than dispersal potential, is a better predictor of the genetic structure across the strait.

### Genetic diversity patterns and refugia within refugia

The phylogeographic arrangement of *A. unedo* does not show the west to east increase in genetic diversity seen in many other plants and animals (Conord *et al*. 2012). Instead, genetic diversity within populations is notably variable and populations with high or low diversity are interspersed all along the species' range. If anything, genetic diversity increases from East to West at a regional level due to the dominance of plants with an Atlantic ancestry in the West and the small number of haplotypes detected in this region.

The predominance of H4-derived haplotypes in FR1 reveals that the Mediterranean lineage managed to reach the Atlantic coast. Interestingly, FR1 shares a private haplotype (H6) with north-east Iberia (SP7) and the Balearics (SP8). Indeed, north-east Iberia (SP7) is more closely related to French demes than to other Iberian populations. Thus, the strawberry tree adds to the many cases of trees with a clear-cut East–West division in the Iberian Peninsula (Rodríguez-Sánchez *et al*. 2010), providing another example of the pattern typically attributed to the ‘refugia-within-refugia’ model (Gómez and Lunt 2007). Since western and eastern Iberia are characterized by different soil types, it has been speculated that soil type might have promoted the isolation of lineages in the peninsula (Rodríguez-Sánchez *et al*. 2010). However, the potential of ecological factors to determine the genetic structure of *A. unedo* is uncertain since common garden experiments show the absence of local adaptation for morphological and physiological traits in this species (Santiso *et al*. 2015).

Finally, our results discard a Tertiary origin for *A. unedo* in Ireland (Sealy 1949). Instead, a relatively recent colonization of Ireland seems a more plausible alternative given (i) the absence of private haplotypes in Ireland and (ii) the genetic proximity between Irish and Iberian population (SAMOVA results). The arrival of the strawberry tree to Ireland has been tentatively attributed to movements along northern Iberia and the maritime fringe of France (Sealy 1949; Cox and Moore 2005). Our results do not necessarily support this hypothesis because none of the haplotypes recorded in Ireland was detected in FR1, at the northern edge of the continuous range of *A. unedo* in the Atlantic. As noted above, FR1 is dominated by haplotypes with a Mediterranean ancestry that are conspicuously absent from Ireland. Moreover, the few H2-derived haplotypes found in FR1 are different from the only H2-derived haplotype detected in Ireland. Alternatively, France could still appear as a plausible source for the Irish populations if the set of haplotypes that currently inhabit FR1 arrived there after the colonization of Ireland. However, this alternative explanation would require a total displacement/disappearance of any previous haplotype in FR1, and we cannot find any likely explanation for such replacement. Hence, our data seem more easily consistent with an Iberian origin for the Irish populations of the strawberry tree. A similar Iberian origin has been proposed for oaks (Kelleher *et al*. 2004; Lowe *et al*. 2005), and the arrival to Ireland by crossing the Celtic Sea has been suggested for other tree species (Mitchell 2006).

## Conclusions

The view that species respond on an individual basis to climate change and create new community patterns has gained increasing support in the last decades (Rodríguez-Sánchez *et al*. 2010). Here, we have shown concordances and discrepancies between the phylogeographic structure of the strawberry tree and that of other plants investigated in the Mediterranean basin. The partition of the strawberry tree into two clades could be attributed to vicariance events during the Quaternary glaciations of the last 700 ky, but before the LGM. These vicariance events possibly occurred in glacial refugia located in the western end of the Mediterranean region. Thus, the Atlantic clade possibly survived in North Africa–Atlantic Iberia, whereas the Mediterranean clade may have resisted in the large islands and the European rim of the Western Mediterranean sub-basin. We also found evidence of occasional connections between Europe and North Africa through the straits of Gibraltar and Sicily, but North Africa still retains a distinctive genetic composition that provides further support to its role as a glacial refugium. From these refugia, the Eastern Mediterranean possibly was colonized more recently, and its lower genetic diversity contrasts with the pattern commonly seen in most plants. The East–West genetic split shown by the strawberry tree in the Iberian Peninsula resembles the pattern found in many other trees and provides a new example of genetic complexity that seems consistent with the refugia-within-refugia model (Gómez and Lunt 2007). Our results discard a Tertiary origin for *A. unedo* in Ireland and do not support a continuous range along the maritime fringe of France either. Instead, the strawberry tree may have arrived to Ireland in post-glacial times from N Iberia by crossing the Celtic Sea. Altogether, our results reveal a considerable ability to disperse for the strawberry tree, migrating over thousands of kilometres and even crossing stretches of sea (Mediterranean straits). This ability for long-distance migration could be useful in a scenario of future changes, allowing the species to migrate to new areas such as Northern France, where a drier and warmer climate, with a greater number of extreme weather events, it is expected (De Vries *et al*. 2012; Habets *et al*. 2013).

## Accession Numbers

These sequences can be obtained from GenBank using the accession numbers: BankIt 1868954: KU 194976–KU195222 (Intron L fragment); BankIt 1874447: KU205359–KU205584 (trnT-tnrL fragment); BankIt 1874452: KU205585–KU205820 (trnL-rpl32 fragment); BankIt 1874463: KU205821–KU206025 (trnH-psbA fragment).

## Sources of Funding

This work was supported by the Spanish Ministry of Science and Innovation (research grant CGL2009-11356), the European Regional Development's Fund (ERDF) and also by the Spanish Ministry of Education (FPU fellowship AP-2009-0962 to X.S.).

## Contributions by the Authors

R.R. and R.B. conceived the ideas; X.S. and R.R. collected the data; X.S. and L.L. performed the work on laboratory; X.S., L.L. and R.B. analysed the data and X.S., L.L., R.B. and R.R. led the writing.

## Conflict of Interest Statement

None declared.

## Supporting Information

The following additional information is available in the online version of this article –

**Figure S1.** The variable positions in the 28 haplotypes detected in *A. unedo*. The first 18 haplotypes belong to the Atlantic clade and the last 10 to the Mediterranean one.

**Figure S2.** The genealogy of the haplotypes obtained with FigTree, which visualized the analysis made with BEAST and TreeAnotator. We observed two main clades, the upper one (Atlantic) with 17 haplotypes and the lower one (Mediterranean) with 11 haplotypes.

**Figure S3.** The genealogy of the haplotypes obtained with TCS. They are grouped into three clades, being the ancestral haplotype of each clade represented inside the rectangle.

**Table S1.** The number of individuals per haplotype in 23 populations of *A. unedo*.

Additional Information

## References

[PLW003C1] Avise JC 2004 What is the field of biogeography, and where is it going? Taxon 53:893–898. 10.2307/4135555

[PLW003C2] Avise JC 2009 Phylogeography: retrospect and prospect. Journal of Biogeography 36:3–15. 10.1111/j.1365-2699.2008.02032.x

[PLW003C3] Avise JC, Ball RM 1990 Principles of genealogical concordance in species concepts and biological taxonomy. Oxford Surveys in Evolutionary Biology 7:45–67.

[PLW003C4] Axelrod DI 1975 Evolution and biogeography of madrean-tethyan sclerophyll vegetation. Annals of the Missouri Botanical Garden 62:280–334. 10.2307/2395199

[PLW003C5] Bandelt HJ, Forster P, Röhl A 1999 Median-joining networks for inferring intraspecific phylogenies. Molecular Biology and Evolution 16:37–48. 10.1093/oxfordjournals.molbev.a026036 10331250

[PLW003C6] Beatty GE, Provan J 2013 Post-glacial dispersal, rather than in situ glacial survival, best explains the disjunct distribution of the Lusitanian plant species *Daboecia cantabrica* (Ericaceae). Journal of Biogeography 40:335–344. 10.1111/j.1365-2699.2012.02789.x

[PLW003C7] Beatty GE, Provan J 2014 Phylogeographical analysis of two cold-tolerant plants with disjunct Lusitanian distributions does not support in situ survival during the last glaciation. Journal of Biogeography 41:2185–2193. 10.1111/jbi.12371

[PLW003C8] Bouckaert R, Heled J, Kühnert D, Vaughan T, Wu CH, Xie D, Suchard MA, Rambaut A, Drummond AJ 2014 BEAST 2: a software platform for Bayesian evolutionary analysis. PLoS Computational Biology 10:e1003537 10.1371/journal.pcbi.1003537 24722319 PMC3985171

[PLW003C9] Bucci G, González-Martínez SC, Le Provost G, Plomion C, Ribeiro MM, Sebastiani F, Alía R, Vendramin GG 2007 Range-wide phylogeography and gene zones in *Pinus pinaster* Ait. revealed by chloroplast microsatellite markers. Molecular Ecology 16:2137–2153. 10.1111/j.1365-294X.2007.03275.x 17498237

[PLW003C10] Casimiro-Soriguer R, Talavera M, Balao F, Terrab A, Herrera J, Talavera S 2010 Phylogeny and genetic structure of *Erophaca* (Leguminosae), a East–West Mediterranean disjunct genus from the Tertiary. Molecular Phylogenetics and Evolution 56:441–450. 10.1016/j.ympev.2010.02.025 20219686

[PLW003C11] Clausing G, Vickers K, Kadereit JW 2000 Historical biogeography in a linear system: genetic variation of Sea Rocket (*Cakile maritima*) and Sea Holly (*Eryngium maritimum*) along European coasts. Molecular Ecology 9:1823–1833. 10.1046/j.1365-294x.2000.01083.x 11091318

[PLW003C12] Clement M, Posada D, Crandall KA 2000 TCS: a computer program to estimate gene genealogies. Molecular Ecology 9:1657–1659. 10.1046/j.1365-294x.2000.01020.x 11050560

[PLW003C13] Conord C, Gurevitch J, Fady B 2012 Large-scale longitudinal gradients of genetic diversity: a meta-analysis across six phyla in the Mediterranean basin. Ecology and Evolution 2:2600–2614. 10.1002/ece3.350 23145344 PMC3492785

[PLW003C14] Cox C, Moore PD 2005 Biogeography: an ecological and evolutionary approach, 7th edn Oxford, UK: Wiley-Blackwell.

[PLW003C15] Darriba D, Taboada GL, Doallo R, Posada D 2012 jModelTest 2: more models, new heuristics and parallel computing. Nature Methods 9:772 10.1038/nmeth.2109 PMC4594756 22847109

[PLW003C16] Davis B, Brewer S, Stevenson A, Guiot J 2003 The temperature of Europe during the Holocene reconstructed from pollen data. Quaternary Science Reviews 22:1701–1716. 10.1016/S0277-3791(03)00173-2

[PLW003C17] Désamoré A, Laenen B, Devos N, Popp M, González-Mancebo JM, Carine MA, Vanderpoorten A 2011 Out of Africa: north-westwards Pleistocene expansions of the heather *Erica arborea*. Journal of Biogeography 38:164–176. 10.1111/j.1365-2699.2010.02387.x

[PLW003C18] De Vries H, Haarsma RJ, Hazeleger W 2012 Western European cold spells in current and future climate. Geophysical Research Letters 39:L04706 10.1029/2011GL050665

[PLW003C19] Dupanloup I, Schneider S, Excoffier L 2002 A simulated annealing approach to define the genetic structure of populations. Molecular Ecology 11:2571–2581. 10.1046/j.1365-294X.2002.01650.x 12453240

[PLW003C20] Escudero M, Vargas P, Arens P, Ouborg NJ, Luceño M 2010 The east-west-north colonization history of the Mediterranean and Europe by the coastal plant *Carex extensa* (Cyperaceae). Molecular Ecology 19:352–370. 10.1111/j.1365-294X.2009.04449.x 20002603

[PLW003C21] Excoffier L, Lischer HEL 2010 Arlequin suite ver 3.5: a new series of programs to perform population genetics analyses under Linux and Windows. Molecular Ecology Resources 10:564–567. 10.1111/j.1755-0998.2010.02847.x 21565059

[PLW003C22] Fazekas AJ, Steeves R, Newmaster SG 2010 Improving sequencing quality from PCR products containing long mononucleotide repeats. Biotechniques 48:277–285. 10.2144/000113369 20569204

[PLW003C23] Fernández-Mazuecos M, Vargas P 2010 Ecological rather than geographical isolation dominates Quaternary formation of Mediterranean *Cistus* species. Molecular Ecology 19:1381–1395. 10.1111/j.1365-294X.2010.04549.x 20196815

[PLW003C24] Foss P, Doyle G, Nelson E 1987 The distribution of *Erica erigena* R. Ross in Ireland. Watsonia 16:311–327.

[PLW003C25] Fu Y-X 1997 Statistical tests of neutrality of mutations against population growth, hitchhiking and background selection. Genetics 147:915–925. 9335623 10.1093/genetics/147.2.915 PMC1208208

[PLW003C26] Gómez A, Lunt DH 2007 Refugia within refugia: patterns of phylogeographic concordance in the Iberian Peninsula. In: Weiss S, Ferrand N, eds. Phylogeography of southern European refugia. The Netherlands: Springer.

[PLW003C27] Habets F, Boé J, Déqué M, Ducharne A, Gascoin S, Hachour A, Martin E, Pagé C, Sauquet E, Terray L, Thiéry D, Oudin L, Viennot P 2013 Impact of climate change on the hydrogeology of two basins in northern France. Climatic Change 121:771–785. 10.1007/s10584-013-0934-x

[PLW003C28] Hampe A, Arroyo J, Jordano P, Petit RJ 2003 Rangewide phylogeography of a bird-dispersed Eurasian shrub: contrasting Mediterranean and temperate glacial refugia. Molecular Ecology 12:3415–3426. 10.1046/j.1365-294X.2003.02006.x 14629356

[PLW003C29] Hewitt G 1999 Post-glacial re-colonization of European biota. Biological Journal of the Linnean Society 68:87–112. 10.1111/j.1095-8312.1999.tb01160.x

[PLW003C30] Hewitt G 2000 The genetic legacy of the Quaternary ice ages. Nature 405:907–913. 10.1038/35016000 10879524

[PLW003C31] Hewitt G 2011 Mediterranean peninsulas: the evolution of hotspots. In: Zachos FE, Habel JC, eds. Biodiversity hotspots. Berlin: Springer.

[PLW003C32] Hileman LC, Vasey MC, Parker VT 2001 Phylogeny and biogeography of the Arbutoideae (Ericaceae): implications for the Madrean-Tethyan hypothesis. Systematic Botany 26:131–143.

[PLW003C33] Huang C-C, Hung K-H, Wang WK, Ho CW, Huang CL, Hsu TW, Osada N, Hwang CC, Chiang TY 2012 Evolutionary rates of commonly used nuclear and organelle markers of *Arabidopsis* relatives (Brassicaceae). Gene 499:194–201. 10.1016/j.gene.2012.02.037 22426291

[PLW003C34] Kadereit G, Yaprak AE 2008 *Microcnemum coralloides* (Chenopodiaceae-Salicornioideae): an example of intraspecific East-West disjunctions in the Mediterranean region. Anales del Jardín Botánico de Madrid 65:415–426.

[PLW003C35] Kadereit JW, Arafeh R, Somogyi G, Westberg E 2005 Terrestrial growth and marine dispersal? Comparative phylogeography of five coastal plant species at a European scale. Taxon 54:861–876. 10.2307/25065473

[PLW003C36] Kelleher C, Hodkinson T, Kelly D, Douglas G 2004 Characterisation of chloroplast DNA haplotypes to reveal the provenance and genetic structure of oaks in Ireland. Forest Ecology and Management 189:123–131. 10.1016/j.foreco.2003.07.032

[PLW003C37] Kropf M, Kadereit JW, Comes HP 2002 Late Quaternary distributional stasis in the submediterranean mountain plant *Anthyllis montana* L. (Fabaceae) inferred from ITS sequences and amplified fragment length polymorphism markers. Molecular Ecology 11:447–463. 10.1046/j.1365-294X.2002.01446.x 11918780

[PLW003C38] Librado P, Rozas J 2009 DnaSP v5: a software for comprehensive analysis of DNA polymorphism data. Bioinformatics 25:1451–1452. 10.1093/bioinformatics/btp187 19346325

[PLW003C39] López de Heredia U, Carrión JS, Jiménez P, Collada C, Gil L 2007 Molecular and palaeoecological evidence for multiple glacial refugia for evergreen oaks on the Iberian Peninsula. Journal of Biogeography 34:1505–1517. 10.1111/j.1365-2699.2007.01715.x

[PLW003C40] Lowe A, Unsworth C, Gerber S, Davies S, Munro R, Kelleher C, King A, Brewer S, White A, Cottrell J 2005 Route, speed and mode of oak postglacial colonisation across the British Isles: integrating molecular ecology, palaeoecology and modelling approaches. Botanical Journal of Scotland 57:59–81. 10.1080/03746600508685085

[PLW003C41] Lumaret R, Mir C, Michaud H, Raynal V 2002 Phylogeographical variation of chloroplast DNA in holm oak (*Quercus ilex* L.). Molecular Ecology 11:2327–2336. 10.1046/j.1365-294X.2002.01611.x 12406243

[PLW003C42] Lumaret R, Tryphon-Dionnet M, Michaud H, Sanuy A, Ipotesi E, Born C, Mir C 2005 Phylogeographical variation of chloroplast DNA in cork oak (*Quercus suber*). Annals of Botany 96:853–861. 10.1093/aob/mci237 16103038 PMC4247051

[PLW003C43] Martínez-Alberola F, Del Campo EM, Lázaro-Gimeno D, Mezquita-Claramonte S, Molins A, Mateu-Andrés I, Pedrola-Monfort J, Casano LM, Barreno E 2013 Balanced gene losses, duplications and intensive rearrangements led to an unusual regularly sized genome in *Arbutus unedo* chloroplasts. PLoS ONE 8:e79685 10.1371/journal.pone.0079685 24260278 PMC3832540

[PLW003C44] Médail F, Diadema K 2009 Glacial refugia influence plant diversity patterns in the Mediterranean Basin. Journal of Biogeography 36:1333–1345. 10.1111/j.1365-2699.2008.02051.x

[PLW003C45] Migliore J, Baumel A, Juin M, Médail F 2012 From Mediterranean shores to central Saharan mountains: key phylogeographical insights from the genus *Myrtus*. Journal of Biogeography 39:942–956. 10.1111/j.1365-2699.2011.02646.x

[PLW003C46] Mitchell FJG 1993 The biogeographical implications of the distribution and history of the strawberry tree, *Arbutus unedo* in Ireland. In: Costello MJ, Kelly KS, eds. Biogeography of Ireland: past, present, and future. Dublin: Irish Biogeographical Society.

[PLW003C47] Mitchell FJG 2006 Where did Ireland's trees come from? Biology & Environment: Proceedings of the Royal Irish Academy 106:251–259. 10.3318/BIOE.2006.106.3.251

[PLW003C48] Myers N, Mittermeier RA, Mittermeier CG, Da Fonseca GAB, Kent J 2000 Biodiversity hotspots for conservation priorities. Nature 403:853–858. 10.1038/35002501 10706275

[PLW003C49] Nei M 1987 *Molecular evolutionary**genetics*. New York: Columbia University Press.

[PLW003C50] Nieto Feliner G 2014 Patterns and processes in plant phylogeography in the Mediterranean Basin. A review. Perspectives in Plant Ecology, Evolution and Systematics 16:265–278. 10.1016/j.ppees.2014.07.002

[PLW003C51] Palamarev E 1989 Paleobotanical evidences of the Tertiary history and origin of the Mediterranean sclerophyll dendroflora. Plant Systematics and Evolution 162:93–107. 10.1007/BF00936912

[PLW003C52] Petit RJ, Hampe A, Cheddadi R 2005 Climate changes and tree phylogeography in the Mediterranean. Taxon 54:877–885. 10.2307/25065474

[PLW003C53] Peyron O, Magny M, Goring S, Joannin S, Beaulieu JD 2012 Pollen-inferred quantitative reconstruction of the Holocene climate in the central Mediterranean area (Italy). Geophysical Research Abstracts 14:7377.

[PLW003C54] Polzin T, Daneshmand SV 2003 On Steiner trees and minimum spanning trees in hypergraphs. Operations Research Letters 31:12–20. 10.1016/S0167-6377(02)00185-2

[PLW003C55] Pons O, Petit RJ 1996 Measuring and testing genetic differentiation with ordered *versus* unordered alleles. Genetics 144:1237–1245. 8913764 10.1093/genetics/144.3.1237 PMC1207615

[PLW003C56] Quézel P, Médail F 2003 Ecologie et biogéographie des forêts du bassin méditerranéen. Paris: Elsevier.

[PLW003C57] Rodríguez-Sánchez F, Pérez-Barrales R, Ojeda F, Vargas P, Arroyo J 2008 The Strait of Gibraltar as a melting pot for plant biodiversity. Quaternary Science Reviews 27:2100–2117. 10.1016/j.quascirev.2008.08.006

[PLW003C58] Rodríguez-Sánchez F, Guzmán B, Valido A, Vargas P, Arroyo J 2009 Late Neogene history of the laurel tree (*Laurus* L., Lauraceae) based on phylogeographical analyses of Mediterranean and Macaronesian populations. Journal of Biogeography 36:1270–1281. 10.1111/j.1365-2699.2009.02091.x

[PLW003C59] Rodríguez-Sánchez F, Hampe A, Jordano P, Arroyo J 2010 Past tree range dynamics in the Iberian Peninsula inferred through phylogeography and palaeodistribution modelling: a review. Review of Palaeobotany and Palynology 162:507–521. 10.1016/j.revpalbo.2010.03.008

[PLW003C60] Santiso X, López L, Gilbert KJ, Barreiro R, Whitlock MC, Retuerto R 2015 Patterns of genetic variation within and among populations in *Arbutus unedo* and its relation with selection and evolvability. Perspectives in Plant Ecology, Evolution and Systematics 17:185–192. 10.1016/j.ppees.2015.02.006

[PLW003C61] Sealy JR 1949 Arbutus unedo. The Journal of Ecology 37:365–388. 10.2307/2256613

[PLW003C62] Shaw J, Lickey EB, Beck JT, Farmer SB, Liu W, Miller J, Siripun KC, Winder CT, Schilling EE, Small RL 2005 The tortoise and the hare II: relative utility of 21 noncoding chloroplast DNA sequences for phylogenetic analysis. American Journal of Botany 92:142–166. 10.3732/ajb.92.1.142 21652394

[PLW003C63] Shaw J, Lickey EB, Schilling EE, Small RL 2007 Comparison of whole chloroplast genome sequences to choose noncoding regions for phylogenetic studies in angiosperms: the tortoise and the hare III. American Journal of Botany 94:275–288. 10.3732/ajb.94.3.275 21636401

[PLW003C64] Smith RJ, Waldren S 2010 Patterns of genetic variation in *Colchicum autumnale* L. and its conservation status in Ireland: a broader perspective on local plant conservation. Conservation Genetics 11:1351–1361. 10.1007/s10592-009-9964-3

[PLW003C65] Smith SA, Donoghue MJ 2008 Rates of molecular evolution are linked to life history in flowering plants. Science 322:86–89. 10.1126/science.1163197 18832643

[PLW003C66] Stewart JR, Lister AM, Barnes I, Dalén L 2010 Refugia revisited: individualistic responses of species in space and time. Proceedings of the Royal Society B: Biological Sciences 277:661–671. 10.1098/rspb.2009.1272 PMC2842738 19864280

[PLW003C67] Taberlet P, Gielly L, Pautou G, Bouvet J 1991 Universal primers for amplification of three non-coding regions of chloroplast DNA. Plant Molecular Biology 17:1105–1109. 10.1007/BF00037152 1932684

[PLW003C68] Torres JA, Valle F, Pinto C, García-Fuentes A, Salazar C, Cano E 2002 *Arbutus unedo* L. communities in southern Iberian Peninsula mountains. Plant Ecology 160:207–223. 10.1023/A:1015864821706

[PLW003C69] Van Andel TH 2002 The climate and landscape of the middle part of the Weichselian glaciation in Europe: the stage 3 project. Quaternary Research 57:2–8. 10.1006/qres.2001.2294

[PLW003C70] Van Rijn P 2004 The analysis of charcoal from Ross Island. In: O'brien W, ed. Ross Island. Mining, metal and society in early Ireland. Bronze age studies 6 Galway: National University of Ireland.

[PLW003C71] Webb DA 1983 The flora of Ireland in its European context. Journal of Life Sciences, Royal Dublin Society 4:143–160.

