# Supplementary material for: Phylogeography of a widespread species: pre-glacial vicariance, refugia, occasional blocking straits and long-distance migrations
Source: AoB Plants. 2016 Jan 14;8:plw003. doi: 10.1093/aobpla/plw003 (PMC4768523; doi:10.1093/aobpla/plw003)
Supplement: Additional Information [file supp_8_plw003_index.html]

Additional Information 

# Phylogeography of a widespread species: pre-glacial vicariance, refugia, occasional blocking straits and long-distance migrations

## Additional Information

Additional Information

- Supplementary Figures - docx file
- Supplementary Table 1 - docx file
