# Supplementary material for: Phylogeography of a widespread species: pre-glacial vicariance, refugia, occasional blocking straits and long-distance migrations
Source: AoB Plants. 2016 Jan 14;8:plw003. doi: 10.1093/aobpla/plw003 (PMC4768523; doi:10.1093/aobpla/plw003)
Supplement: Additional Information [file supp_plw003_plw003supp_figures.docx]

**SUPPORTING INFORMATION**

**
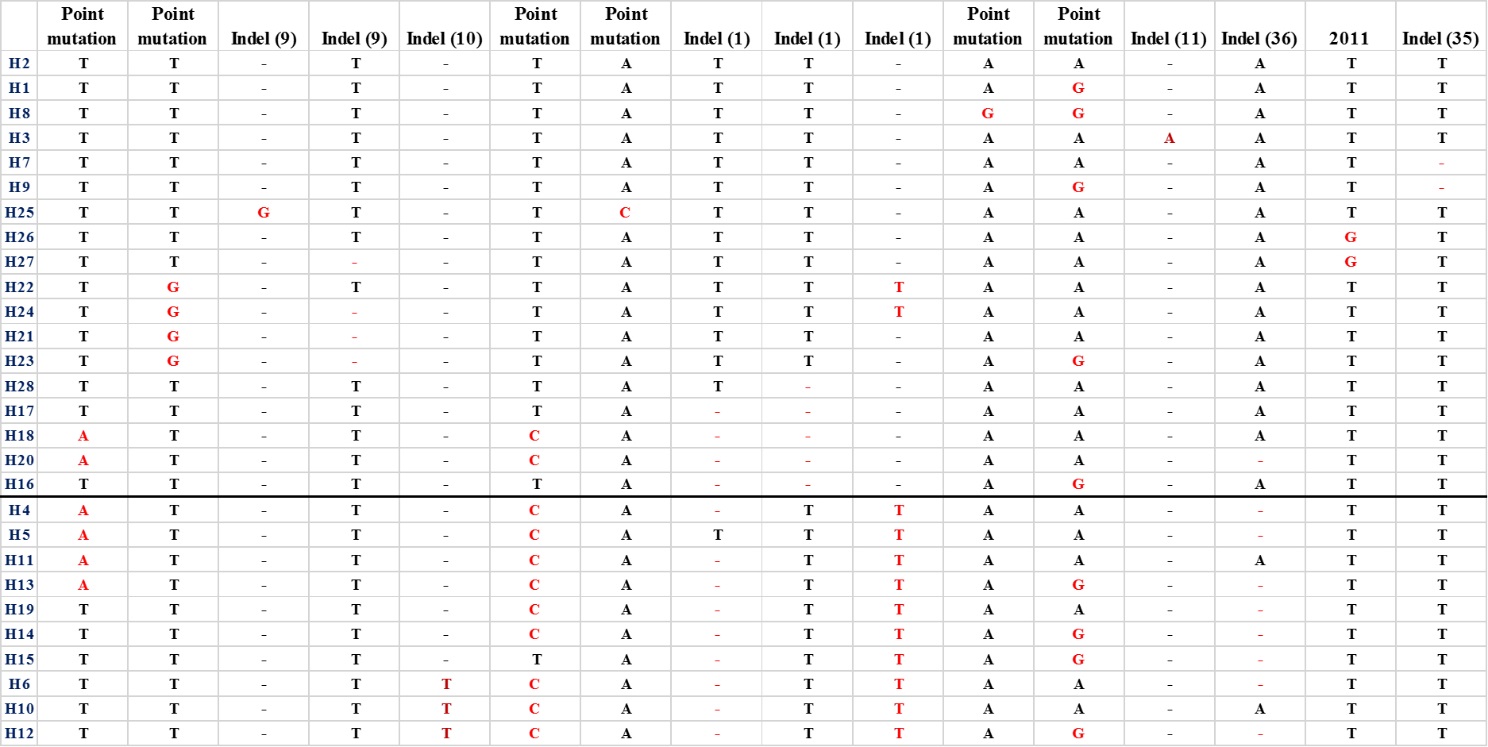
**

**Figure S1.** Variable positions in the 28 haplotypes detected in Arbutus unedo. The first 18 haplotypes belong to the Atlantic clade, the last 10 to the Mediterranean one.

**
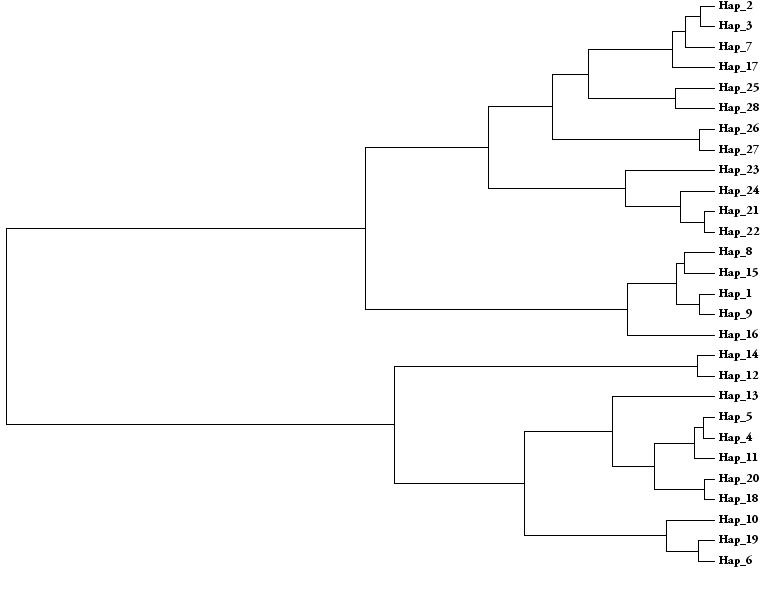
**

**Figure S2.** Genealogy of the haplotypes obtained with FigTree, which visualized the analysis made with BEAST and TreeAnotator. We observed two main clades, the upper one (Atlantic) with 17 haplotypes and the lower (Mediterranean) with 11 haplotypes.

**
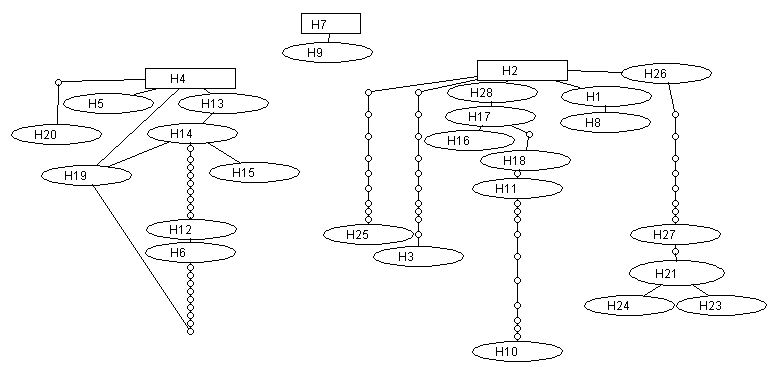
**

**Figure S3**. Genealogy of the haplotypes obtained with TCS. Three clades are observed, being the ancestral haplotype of each clade represented inside the rectangle. H7-H9 clade is closest to H2 clade, and both together are the Atlantic group. H4 clade constitute the Mediterranean group.
