# Supplementary material for: Phylogeography of a widespread species: pre-glacial vicariance, refugia, occasional blocking straits and long-distance migrations
Source: AoB Plants. 2016 Jan 14;8:plw003. doi: 10.1093/aobpla/plw003 (PMC4768523; doi:10.1093/aobpla/plw003)
Supplement: Additional Information [file supp_plw003_plw003supp_table_1.docx]

Table S1. Number of individuals per haplotype in 23 population of *A. unedo*. Population codes as in Fig. 1. The population codes used in GenBank are also showed.

| Pop | GenBank  Codes | Atlantic clade | | | | | | | | | | | | | | | | | | | Mediterranean clade | | | | | | | | | |  |
| --- | --- | --- | --- | --- | --- | --- | --- | --- | --- | --- | --- | --- | --- | --- | --- | --- | --- | --- | --- | --- | --- | --- | --- | --- | --- | --- | --- | --- | --- | --- | --- |
|  |  | H2 | H1 | H8 | H3 | H7 | H9 | H25 | H26 | H27 | H22 | H24 | H21 | H23 | H28 | H17 | H18 | H20 | H16 | H4 | | H5 | H11 | H13 | H19 | H14 | H15 | H6 | H10 | H12 | **Total** |
| IRL | IK |  |  |  |  | 5 | 7 |  |  |  |  |  |  |  |  |  |  |  |  |  | |  |  |  |  |  |  |  |  |  | **12** |
| SP1 | EF |  | 1 | 3 |  |  | 2 |  |  |  |  |  |  |  |  |  |  |  |  |  | |  |  |  |  |  |  |  |  |  | **6** |
| SP2 | EE | 3 | 2 | 1 |  | 1 |  |  |  |  |  |  |  |  |  |  |  |  |  |  | |  |  |  |  |  |  |  |  |  | **7** |
| SP3 | EA | 1 | 4 |  | 1 |  |  |  |  |  |  |  |  |  |  |  |  |  |  |  | |  |  |  |  |  |  |  |  |  | **6** |
| SP4 | ET | 3 |  | 3 |  |  |  |  |  |  |  |  |  |  |  |  |  |  |  |  | |  |  |  |  |  |  |  |  |  | **6** |
| SP5 | EP | 1 |  | 1 |  | 8 |  |  |  |  |  |  |  |  |  |  |  |  |  |  | |  |  |  |  |  |  |  |  |  | **10** |
| PO1 | PA | 5 |  |  |  | 1 |  |  | 4 | 1 |  |  |  |  |  |  |  |  |  |  | |  |  |  |  |  |  |  |  |  | **11** |
| PO2 | PM | 3 |  |  |  | 7 |  |  |  |  |  |  |  |  |  |  |  |  |  |  | |  |  |  |  |  |  |  |  |  | **10** |
| SP6 | EM | 12 |  |  |  |  |  |  |  |  |  |  |  |  |  |  |  |  |  |  | |  |  |  |  |  |  |  |  |  | **12** |
| MO1 | MT | 9 |  |  |  |  |  | 2 |  |  |  |  |  |  |  |  |  |  |  |  | |  |  |  |  |  |  |  |  |  | **11** |
| MO2 | MD | 1 |  |  |  |  |  |  |  |  | 1 | 1 | 4 | 1 |  |  |  |  |  |  | |  |  |  |  |  |  |  |  |  | **8** |
| TUN | TK | 2 |  |  |  |  |  |  |  |  |  |  |  |  |  |  |  |  |  | 3 | | 1 |  | 1 |  |  |  |  |  |  | **7** |
| SP7 | EC |  |  |  |  |  |  |  |  |  |  |  |  |  |  |  |  |  |  | 7 | | 1 |  |  |  |  |  | 2 |  |  | **10** |
| SP8 | EI |  |  |  |  |  |  |  |  |  |  |  |  |  |  |  |  |  |  | 4 | |  | 1 |  |  |  |  | 1 | 1 |  | **7** |
| FR1 | FB | 1 | 1 |  |  |  |  |  |  |  |  |  |  |  |  |  |  |  |  |  | |  |  |  |  |  |  | 9 |  | 1 | **12** |
| FR2 | FM |  |  |  |  |  |  |  |  |  |  |  |  |  |  |  |  |  |  | 4 | |  |  | 4 |  | 2 | 1 |  |  |  | **11** |
| IT1 | IT |  |  |  |  |  |  |  |  |  |  |  |  |  |  |  |  |  |  | 4 | |  | 4 |  |  |  |  |  |  |  | **8** |
| IT2 | IR |  |  |  |  |  |  |  |  |  |  |  |  |  |  |  |  | 1 |  | 5 | |  |  |  | 4 |  |  |  |  |  | **10** |
| IT3 | IC |  |  |  |  |  |  |  |  |  |  |  |  |  |  | 2 |  |  | 1 | 6 | |  |  |  |  |  |  |  |  |  | **9** |
| GR1 | GA |  |  |  |  |  |  |  |  |  |  |  |  |  |  | 5 |  |  | 3 |  | |  |  |  |  | 1 |  |  |  |  | **9** |
| GR2 | GS |  |  |  |  |  |  |  |  |  |  |  |  |  |  | 6 | 1 |  | 1 |  | |  |  |  |  |  |  |  |  |  | **8** |
| TU1 | TC |  |  |  |  |  |  |  |  |  |  |  |  |  | 3 | 2 |  |  |  |  | |  |  |  | 3 |  |  |  |  |  | **8** |
| TU2 | TI |  |  |  |  |  |  |  |  |  |  |  |  |  |  | 5 |  |  | 1 | 3 | |  |  |  |  |  |  |  |  |  | **9** |
| **Total** |  | **41** | **8** | **8** | **1** | **22** | **9** | **2** | **4** | **1** | **1** | **1** | **4** | **1** | **3** | **20** | **1** | **1** | **6** | **36** | | **2** | **5** | **5** | **7** | **3** | **1** | **12** | **1** | **1** | **207** |
